# B1-insensitive T2 mapping of healthy thigh muscles using a T2-prepared 3D TSE sequence

**DOI:** 10.1371/journal.pone.0171337

**Published:** 2017-02-14

**Authors:** Elisabeth Klupp, Dominik Weidlich, Sarah Schlaeger, Thomas Baum, Barbara Cervantes, Marcus Deschauer, Hendrik Kooijman, Ernst J. Rummeny, Claus Zimmer, Jan S. Kirschke, Dimitrios C. Karampinos

**Affiliations:** 1 Institut für Diagnostische und Interventionelle Neuroradiologie, Technische Universität München, Munich, Germany; 2 Institut für Diagnostische und Interventionelle Radiologie, Technische Universität München, Munich, Germany; 3 Neurologische Klinik und Poliklinik, Technische Universität München, Munich, Germany; 4 Philips Healthcare, Hamburg, Germany; Semmelweis Egyetem, HUNGARY

## Abstract

**Purpose:**

To propose a T2-prepared 3D turbo spin echo (T2prep 3D TSE) sequence for B1-insensitive skeletal muscle T2 mapping and compare its performance with 2D and 3D multi-echo spin echo (MESE) for T2 mapping in thigh muscles of healthy subjects.

**Methods:**

The performance of 2D MESE, 3D MESE and the proposed T2prep 3D TSE in the presence of transmit B1 and B0 inhomogeneities was first simulated. The thigh muscles of ten young and healthy subjects were then scanned on a 3 T system and T2 mapping was performed using the three sequences. Transmit B1-maps and proton density fat fraction (PDFF) maps were also acquired. The subjects were scanned three times to assess reproducibility. T2 values were compared among sequences and their sensitivity to B1 inhomogeneities was compared to simulation results. Correlations were also determined between T2 values, PDFF and B1.

**Results:**

The left rectus femoris muscle showed the largest B1 deviations from the nominal value (from 54.2% to 92.9%). Significant negative correlations between T2 values and B1 values were found in the left rectus femoris muscle for 3D MESE (r = -0.72, *p*<0.001) and 2D MESE (r = -0.71, *p*<0.001), but not for T2prep 3D TSE (r = -0.32, *p* = 0.09). Reproducibility of T2 expressed by root mean square coefficients of variation (RMSCVs) were equal to 3.5% in T2prep 3D TSE, 2.6% in 3D MESE and 2.4% in 2D MESE. Significant differences between T2 values of 3D sequences (T2prep 3D TSE and 3D MESE) and 2D MESE were found in all muscles with the highest values for 2D MESE (*p*<0.05). No significant correlations were found between PDFF and T2 values.

**Conclusion:**

A strong influence of an inhomogeneous B1 field on the T2 values of 3D MESE and 2D MESE was shown, whereas the proposed T2prep 3D TSE gives B1-insensitive and reproducible thigh muscle T2 mapping.

## Introduction

Acute inflammatory edematous alterations of skeletal muscles, reflecting disease activity, as well as fatty infiltration of chronically affected muscles are two main characteristics of neuromuscular diseases [1–3]. Conventional diagnostic magnetic resonance imaging (MRI), based on T1-weighted, T2-weighted and Short Tau Inversion Recovery (STIR) sequences, is only able to detect qualitative pathological changes in the muscle tissue. Semi-quantitative scales exist for judging the extent of fatty infiltration or edematous muscular alterations based on conventional MR images [4–6]. However, the evaluation of conventional images can become strongly dependent on the reader’s judgment. There is therefore an emerging need for objective evaluation of fatty infiltration and inflammatory skeletal muscle alterations based on quantitative imaging.

Chemical shift encoding-based water-fat MRI enables the assessment and quantification of the fatty infiltration in muscle tissue using the proton density fat fraction (PDFF) [7, 8]. T2 mapping is a quantitative MRI technique that enables the quantification of inflammatory changes [1–3]. However, most existing T2 mapping techniques are also sensitive to changes in the fat content. Fat suppression has been proposed as a way to at least partially reduce the effect of fat content on the extracted T2 values [9, 10], however with known limitations on totally removing the effect of the fat content from the extracted T2 values [11].

In most clinical settings, T2 is routinely quantified using multi-echo spin-echo (MESE). In addition to fat effects, a major technical challenge in muscle T2 mapping using MESE is the occurrence of stimulated echoes. Stimulated echoes occur because of slice profile effects, transmit B1 calibration errors, transmit B1 inhomogeneity and B0 inhomogeneity [12]. Using 3D MESE instead of 2D MESE removes the slice profile effects. However, transmit B1 inhomogeneity remains an important source of muscle T2 quantification errors especially in the thigh, a region mainly affected in neuromuscular diseases. The sensitivity of MESE T2 mapping methods to transmit B1 inhomogeneity is a well-known problem, extensively studied also in various body parts, using composite refocusing pulses or advanced signal models [13]. Alternative T2 mapping methods have been also proposed. Specifically, pulse sequence designs using a T2-preparation followed by a gradient echo read-out have been recently emerging for T2 mapping in the brain and the myocardium [14, 15], with special focus on the RF pulse design of the T2-preparation to assure B1 insensitivity. T2-preparation followed by a 3D turbo spin echo (3D TSE) read-out has also been also proposed [16] to avoid the T1 contamination effects associated with the gradient echo read-out [17].

An adiabatic BIR-4 radiofrequency (RF) pulse previously used for generating T2-weighted contrast [18] and recently adjusted with gaps (modified BIR-4) [19] for an adiabatic T2-preparation module with variable TE is presently proposed, combined with a 3D TSE read-out and applied for B1-insensitive skeletal muscle T2 quantification. In addition, fat suppression is used to reduce the effect of fat on the extracted T2 values. The purpose of the present work is to compare the proposed T2prep 3D TSE with routinely used 2D and 3D MESE for T2 mapping in thigh muscles of healthy subjects, including range of T2 values, T2 mapping reproducibility and influence of transmit B1-field as well as proton density fat fraction (PDFF) on measured T2 values.

## Materials and methods

### T2prep 3D TSE pulse sequence

**Fig 1:**
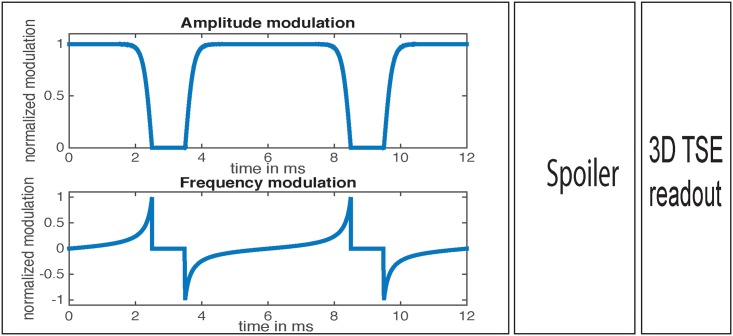
Pulse sequence diagram for T2prep-3DTSE. Pulse sequence diagram for T2prep-3DTSE, showing amplitude modulation (a) and frequency modulation (b) of the modified BIR-4 T2-preparation module.

A T2-preparation pulse sequence module was developed based on a modified BIR-4 RF pulse, where two gaps with equal duration were introduced to achieve a module with variable TE. The T2-preparation module was followed by a spoiler and a 3D TSE read-out. Fig 1 shows the sequence diagram of the T2prep 3D TSE and the timing of the developed T2-preparation module. The parameters of the employed BIR-4 RF pulse were: total pulse duration = 10 ms, B1 amplitude = 13.5 μT and frequency sweep = 3700 Hz.

### Subjects

Ten young and healthy subjects (4 male and 6 female, age: 25.9 ± 3.07 years) were recruited for this study. Inclusion criteria were no history of diabetes, neuromuscular disorders and previous quadriceps muscle injuries. The study was approved by the ethics commission of the medical faculty of the Technical University Munich (TUM). Before participation, all subjects gave written informed consent.

### MRI measurements

The bilateral thigh muscles were scanned on a 3 T system (Ingenia, Philips Healthcare, Best, Netherlands) using anterior and posterior coil arrays. The field of view (FoV) was centered at the femur mid-length. To obtain a reproducible positioning, the greater trochanter and the tibial plateau served as anatomical landmarks and the distance between them was halved, as described by Azzabou et al [20].

T2 mapping was performed using 2D MESE, 3D MESE and T2prep 3D TSE. Spectral Adiabatic Inversion Recovery (SPAIR) was used for suppression of the main fat peaks in all T2 mapping sequences. The common sequence parameters were: FoV = 42×26×8 cm^3^, reconstructed voxel-size = 1.75×1.75×4 mm^3^ and SENSE parallel imaging with reduction factor of 2.

The T2prep 3D TSE had the following parameters: echo spacing = 2.4 ms, acquisition voxel = 2×2×8 mm^3^, echo train length = 50, T2prep durations of 20/30/40/50/60/70 ms and a scan duration of 4:35 min for 20 slices with TR of 1500ms and an effective TE of the TSE shot of 19 ms. The flip angle train of the TSE readout was designed in order to achieve a constant signal plateau throughout the TSE shot (signal plateau = shot defined, with ratio = 1) for skeletal muscle: the flip angles of the TSE readout were ramped down to 24° during the first 5 start-up echoes and subsequently increased to 100° to achieve a constant signal plateau and then linearly increased to 125° [21].

For the MESE-sequences the standard sequence implementations, provided by the MR vendor, were used. In the 2D case 20 slices were acquired in 6:03 min with TR/TE of 1500/10 ms and acquisition voxel of 2×2×4 mm^3^. In the 3D MESE the scan duration was 9:48 min for 10 slices with TR/TE of 1000/10 ms and acquisition voxel of 2×2×8 mm^3^. In both sequences TEs of 20/30/40/50/60/70 ms and 180° refocusing pulses were used.

Additionally, a B1-map was acquired with the dual TR method [22] using a 3D gradient echo sequence with parameters: FOV = 42×26×8 cm^3^, acquired voxel size = 4×4×4 mm^3^, TR1/TR2/TE = 20/100/2.3 ms, flip-angle = 60°. B0- and PDFF-maps were also measured based on a 6-echo 3D gradient echo sequence with parameters: FOV = 42×26×8 cm^3^, acquired voxel size = 3.2×2×4 mm^3^, TR/TE1/ΔTE = 10/1.13/0.8 ms, flip-angle = 3° and six acquired echoes (in a single TR using a bipolar gradient readout).

Bloch simulations were also performed in order to characterize the sensitivity of the employed MESE sequences and the T2prep module to B1 and B0 inhomogeneities for skeletal muscle (T1 = 1420 ms, T2 = 32 ms) [23]. For all three methods TEs of 20/30/40/50/60/70 ms were simulated.

### Data analysis

Post-processing of the T2 maps was performed offline with the same routine for all three sequences in order to maximize comparability. Data was fitted by a 2-parameter fit using linear least-squares and the first echo was excluded for the MESE data to reduce T2 overestimation due to stimulated echoes.

For the B1-map as well as the B0- and PDFF-maps the post-processing routines, provided online by the MR vendor, were used. In the post-processing step of the dual TR method, the ratio of the two acquired signals was used to calculate an effective flip angle. The ratio of the effective and the nominal flip angle determined the B1 field strength. PDFF quantification was performed based on a complex-based water-fat decomposition by using a single T_2_* correction and a pre-calibrated fat spectrum [24, 25], after correcting for phase errors effects. The imaging-based PDFF-map was computed as the ratio of the fat signal over the sum of fat and water signals.

The first and the last slice of the 3D sequences were affected by slab profile effects and therefore excluded from analyses resulting in eight analyzable slices. Two representative muscles (rectus femoris, vastus lateralis) of the left and right quadriceps muscle, mainly affected in neuromuscular disorders, were selected for further analyses. ROIs were drawn manually by two operators in the interior of these muscles avoiding vessels and fasciae (using OsiriX). Volumes of these four muscles as well as the different values were extracted for all sequences.

### Reproducibility and correlation analysis

To assess and compare the reproducibility of the three different T2 mapping sequences, the ten subjects were scanned three times with repositioning. The above mentioned segmentation procedure was performed in all images. Reproducibility errors were expressed as root mean square coefficients of variation (RMSCV) in [%], according to Gluer et al [26].

Measured T2 values were correlated with transmit B1 field values in the left rectus femoris, as this muscle showed the largest transmit B1 errors in the present experimental set-up. Measured T2 values were also correlated with PDFF values in all muscles.

### Statistical tests

The statistical analyses were performed with SPSS 23.0 (SPSS, Chicago, IL, USA). All tests were done using a two-sided level of significance p<0.05. Parameters are presented as mean ± standard deviation (SD). Since the Kolmogorov-Smirnov test partially showed significant differences from normal distribution, correlations between the different MRI-derived parameters were evaluated with the Spearman-Rho correlation coefficient r. t-tests were calculated to compare the different T2 values.

## Results

**Fig 2:**
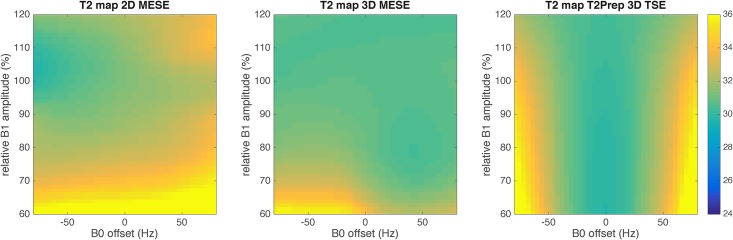
Contour plots showing simulation results of the sensitivity of T2 quantification to B1 and B0 errors. Contour plots showing simulation results of the sensitivity of T2 quantification to B1 and B0 errors for (a) T2prep-3D TSE, (b) 2D MESE and (c) 3D MESE.

Fig 2a shows the simulated large dependency of the 2D MESE sequence on the transmit B1 field for a range of B1 values between 60% and 120%. The deviation from the real T2 value is increased with decreasing B1 field amplitude. In Fig 2b the performance of the 3D MESE sequence is illustrated. The estimated T2 value is also dependent on the B1 field amplitude but the simulated T2 value is stable over a much wider range of possible B1 errors. Fig 2c shows a stable T2 quantification for the employed T2-preparation. The range of B0 errors over which the employed T2-preparation provides a stable T2 quantification decreases as the B1 value decreases (Fig 2c). For B0 offsets between -60 Hz and 60 Hz (typical range for thigh muscle imaging at 3 T), the proposed T2-preparation results in stable T2 quantification for B1 varying in the entire range between 60% and 120%.

**Fig 3:**
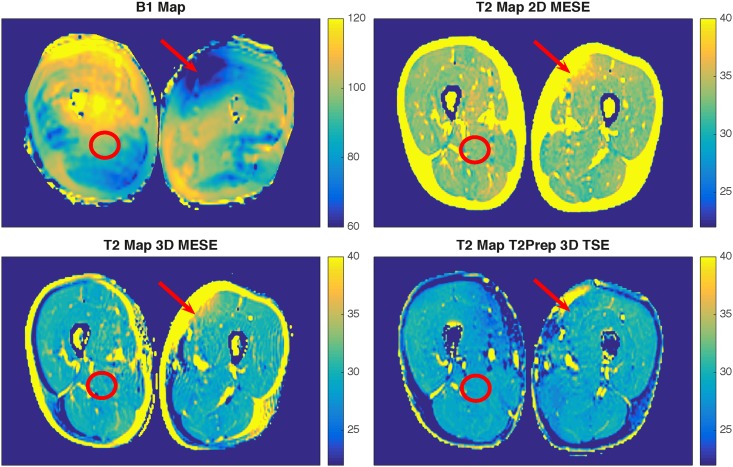
Representative example of B1 map and the three different T2 maps. B1 map (top left) and the three different T2 maps (top right: T2prep 3D TSE, bottom left: 2D MESE, bottom right: 3D MESE) of the thigh musculature of one representative subject. With low B1 (arrow), 2D MESE and 3D MESE are significantly affected. With a small B1 error (circle), 2D MESE shows higher T2 values than 3D MESE and T2prep 3D TSE.

Representative T2 maps generated by the three different T2 mapping sequences and the respective B1 map of one healthy volunteer are shown in Fig 3. When the B1 error was large, T2 values from the MESE-sequences were significantly higher compared to T2prep 3D TSE (arrow). When the B1 error was small, T2 values from 2D MESE were higher than T2 values from 3D MESE and T2prep 3D TSE (circle). Significant differences between the T2 values from the three employed sequences were found in all four muscles with highest T2 values observed when using the 2D MESE sequence (*p*<0.05).

The T2 values assessed by the three T2 mapping sequences, the B0 field and B1 field inhomogeneities and the PDFF of the segmented muscles are shown in Table 1. The left rectus femoris muscle showed the highest inhomogeneity in the B1 field (from 54.2% to 92.9%), compared to the other three studied muscles as well as the highest variance of T2 values. The largest span of B0 was found in the left vastus lateralis muscle (from -34.7% to 86.5%).

**Table 1:** Range of B1, B0, means +/- standard deviations of PDFF and T2 relaxation times for the investigated muscles.

	B1 (range) in %	B0 (range) in %	PDFF (mean±SD) in %	T2prep 3DTSE (mean±SD in ms	3D MESE (mean±SD) in ms	2D MESE (mean±SD) in ms
**Left rectus femoris**	54.2–92.9	-19.4–65.3	4.7±2.8	31.4±1.7	33.3±2.9	36.4±3.1
**Right rectus femoris**	108.6–120.7	-13.6–28.1	3.7±0.9	28.9±1.4	30.2±1.0	33.8±0.9
**Left vastus lateralis**	87.7–108.8	-34.7–86.5	3.8±0.9	31.4±1.4	31.3±1.0	34.3±1.0
**Right vastus lateralis**	85.4–111.7	-39.8–29.2	4.8±3.9	30.9±1.3	30.07±1.0	34.1±1.0

Range of B1 (in %), B0 (in %), means +/- standard deviations of the proton density fat fraction (PDFF; in %) and T2 relaxation times (in ms) using T2prep-3D TSE, 2D MESE and 3D MESE for the four investigated muscles of the thigh region in ten young and healthy subjects.

**Fig 4:**
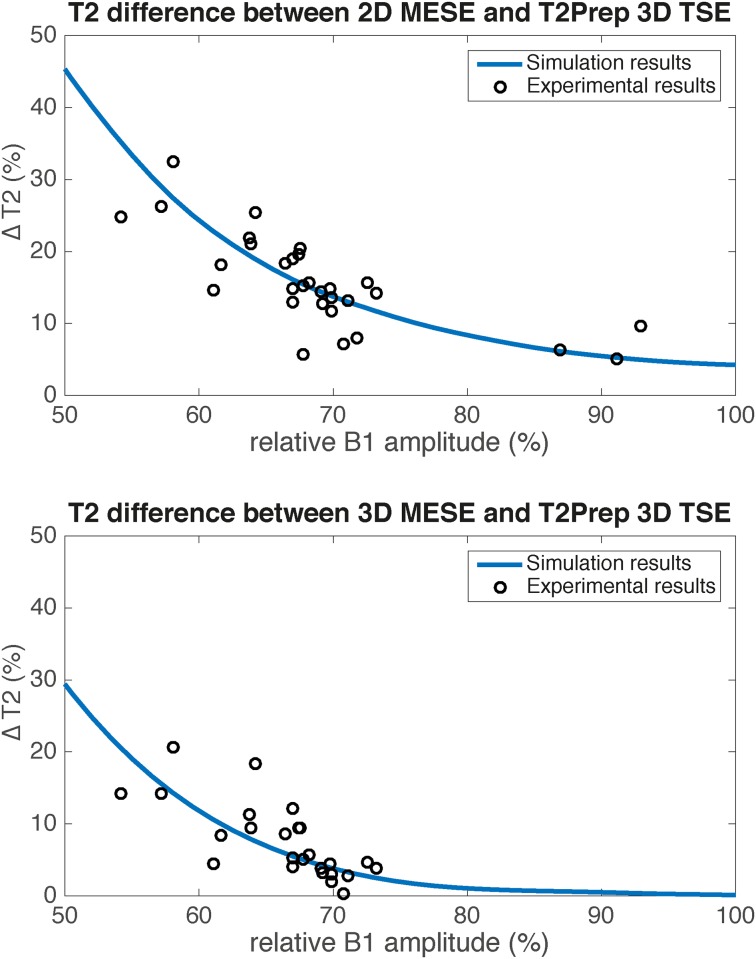
Relative differences (in %) between T2 values as a function of relative B1 amplitude (in %). Relative difference (in %) (a) between T2 values of 2D-MESE and T2 values of T2prep 3D TSE and (b) betweenT2 values of 3D-MESE and T2 values of T2prep 3D TSE as a function of relative B1 amplitude (in %): solid lines show simulation results and points represent experimental results.

In Fig 4 the relative difference between the T2prep 3D TSE and the two MESE sequences is shown for the simulations and the experimental data of the left rectus femoris muscle. For both MESE sequences an increase of the relative T2 difference can be observed when the B1 field is decreasing. The effect was smaller for the 2D than for the 3D MESE sequence. The experimental data show a good agreement with the trend of the simulation and illustrate the dependency of the MESE sequences on B1 errors.

When focusing on the B1 mapping and T2 mapping results for the left rectus femoris muscle of all acquired datasets, a significant negative correlation was found between T2 values and B1 field for 3D MESE (r = -0.72, *p*<0.001) and 2D MESE (r = -0.71, *p*<0.001), but not for T2prep 3D TSE (r = -0.32, *p* = 0.09).

**Fig 5:**
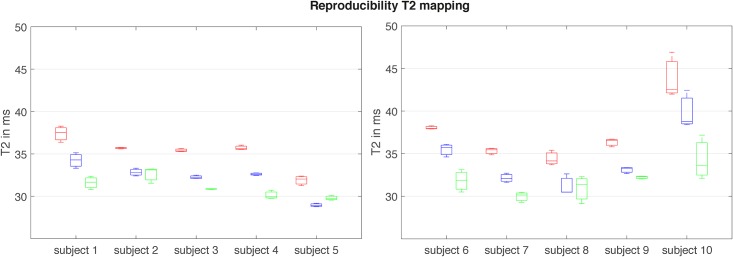
Reproducibility of the T2 values across 10 subjects. Reproducibility of the T2 values assessed by the three different T2mapping methods across 10 scanned subjects in the left rectus femoris muscle. Boxes represent the 25th and 75th percentiles with the median represented by a horizontal line within the box plots. The bars indicate the minimum and maximum. Red: 2D MESE, blue: 3D MESE, green: T2prep 3D TSE.

The assessment of reproducibility revealed the following RMSCVs of the left rectus femoris muscle: T2prep 3D TSE: 3.5%; 3D MESE: 2.6%; 2D MESE: 2.4%. T2 values of the three different T2 mapping sequences are shown in Fig 5. No significant correlations were found between PDFF and T2-values of the different mapping methods.

## Discussion

The present work proposed a newly developed T2 mapping sequence (T2prep 3D TSE) for skeletal muscle T2 mapping and compared the developed T2 mapping sequence with standard 2D and 3D MESE in healthy thigh muscles. The results showed a dependency of the T2 values from 2D MESE and 3D MESE on transmit B1 field, whereas the developed T2prep 3D TSE was less affected by the transmit B1 field and showed a good reproducibility in the thigh musculature of young and healthy subjects.

In the right rectus femoris, left vastus lateralis and right vastus lateralis muscle the transmit B1 error was relatively small (Table 1). In these three muscles, the T2 values based on the 2D MESE were higher than the T2 values from the 3D MESE and T2prep 3D TSE. This degree of T2 overestimation using 2D MESE should be attributed to the occurrence of stimulated echoes due to slice profile effects, which are prominent in 2D imaging but absent in 3D imaging. Due to the clinically non-practical long scan times of the 3D MESE, a good agreement between the T2 values obtained from the T2prep 3D TSE sequence and the 3D MESE sequence is favorable.

The largest B1 errors were reported in the present experimental set-up and volunteer study in the left rectus femoris muscle. Despite the subjects repositioning in the performed reproducibility analysis, B1 errors appeared in a very similar anatomical location independent of repositioning. In the left rectus femoris muscle, 2D MESE as well as 3D MESE resulted in significantly higher T2 values than T2prep 3D TSE, again due to the occurrence of stimulated echoes. Significant negative correlations between left rectus femoris muscle T2 values and B1 values were reported for the two MESE sequences but not for T2prep 3D TSE. In order to remove any effects from intersubject T2 variation in the above correlation analysis, relative T2 differences between the T2prep 3D TSE and the two MESE sequences as a function of the B1 error were also examined. The experimentally measured relative T2 difference between the T2prep 3D TSE and the 2D MESE increased as the B1 field decreased and was in good agreement with the simulation results. A similar trend was also observed between the T2prep 3D TSE and the 3D MESE. The dependence of the relative T2 differences between the T2prep 3D TSE and the two MESE sequences on B1 errors highlighted the dependence of MESE-based T2 quantification on B1 errors.

Removing the B1 sensitivity of T2 mapping is a long-standing problem. The developed T2prep 3DTSE enables B1-insensitive T2 quantification, by ensuring B1 insensitivity on the data acquisition side. An alternative way to remove B1 sensitivity is by modeling the B1 effects on the measured signal. Specifically, recent work from Lebel and Wilman has shown that an application of extended phase graphs (EPG) can compensate for stimulated echoes due to B1 effects in MESE-based quantification of T2 values [13]. Such EPG-based approaches for correcting B1 effects have been also recently implemented in skeletal muscle applications [27, 28].

Another important issue is the influence of fatty infiltration on T2 values. In MESE, both inflammatory alterations as well as fatty transformations, are leading to an increase of the T2-weighted signal due to an increase of the water T2 and/or an increase of the fat content (T2 of fat is longer than T2 of healthy muscle water) [29]. It has already been demonstrated that patients with Duchenne muscular dystrophy showed a significant apparent T2 increase in muscle tissue, mainly caused by fatty infiltration [30, 31], whereas patients with dermatomyositis revealed a significant apparent T2 increase reflecting increased inflammation and edema [32]. In the present work, SPAIR-based fat suppression was employed to reduce the effect of fat on the measured T2 values. Spectrally-selective fat suppression however cannot suppress the olefinic fat peak in the vicinity of the water peak. In addition, fat peaks show J-couplings and have much shorter T1 than water peaks, which further complicate their signal evolution in the presence of stimulated echoes in MESE. In the present study, the T2 values from all methods showed no dependence on the PDFF in the examined thigh muscles, a result that could be attributed to effective fat suppression and the low PDFF value in the studied young and healthy volunteers. However, T2 of the subcutaneous fat region was higher than the T2 of healthy muscle in MESE (Fig 3), whereas T2prep 3DTSE led to T2 values in subcutaneous fat in the same range as in healthy muscle (Fig 3). The observed performance of the developed T2prep 3D TSE in the presence of fat is outside the scope of the present study and would require further investigation.

The present study has some limitations. First, only a relatively small and age-homogeneous subject sample was examined. Second, regarding the comparison of the T2 mapping sequences, there was no real gold standard as both the 2D and 3D MESE were affected by the presence of stimulated echoes. The characterization of the B1 sensitivity of all three investigated T2 mapping sequences was the main aim of the present work. Based on the simulations, the proposed T2prep 3D TSE sequence is minimally affected by B1 inhomogeneities and when used as a reference the experimentally measured relative difference between the T2prep 3D TSE and the MESE techniques showed good agreement with the simulations. Third, since age as well as gender affect the muscle composition [33], the exact results about the range of the extracted T2 values cannot be generalized. Especially, the presence of fat in muscles is an important aspect when comparing T2 mapping methods in muscles of older subjects or of neuromuscular disease patients. However, the B1-insensitivity is a considerable advantage of the developed T2prep 3D TSE, which should be valid across subjects. Fourth, the 2D and 3D MESE sequences were used as provided by the MR vendor without any further optimization of the employed refocusing pulses. Finally, despite the fact that muscle T2 decay is known to be multi-exponential, a mono-exponential model was adopted for simplicity purposes.

## Conclusion

In conclusion, the present work proposed the use of a T2-prepared 3D TSE for robust muscle T2 mapping and was able to show that this method gave B1-insensitive and reproducible T2 values in the thigh muscles of young and healthy volunteers. T2-prepared 3D TSE might therefore be useful as an alternative to standard T2 mapping sequences in settings where strong B1 errors are expected (i.e. in extremities and at high field strengths).

## Supporting information

S1 Table Age, values of B1, B0, PDFF and T2 relaxation times for the investigated muscles. Age (in years), values (means +/- standard deviations) of B1 (in %), B0 (in %), proton density fat fraction (PDFF, in %) and T2 relaxation times (in ms) for the investigated muscles using T2prep-3D TSE, 2D MESE and 3D MESE for the four investigated muscles of the thigh region in ten young and healthy subjects. The values are shown for each scan separately. VLL: left vastus laterais muscle,; VLR: right vastus lateralis muscle; RFL: left rectus femoris muscle; RFR: right rectus femoris muscle.
(PDF)
