# Supplementary material for: B1-insensitive T2 mapping of healthy thigh muscles using a T2-prepared 3D TSE sequence
Source: PLoS One. 2017 Feb 14;12(2):e0171337. doi: 10.1371/journal.pone.0171337 (PMC5308846; doi:10.1371/journal.pone.0171337)
Supplement: S1 Table — Age (in years), values (means +/- standard deviations) of B1 (in %), B0 (in %), proton density fat fraction (PDFF, in %) and T2 relaxation times (in ms) for the investigated muscles using T2prep-3D TSE, 2D MESE and 3D MESE for the four investigated muscles of the thigh region in ten young and healthy subjects. The values are shown for each scan separately. VLL: left vastus laterais muscle,; VLR: right vastus lateralis muscle; RFL: left rectus femoris muscle; RFR: right rectus femoris muscle. (PDF) [file pone.0171337.s001.pdf]

Subject	Age		Muscle	volume (cm^3)	T2prep 3D TSE (ms)	2D MESE (ms)	3D MESE (ms)	B1 (%)	B0 (%)	Fat Fraction (%)
**1**	29	scan 1								
			RFL	6.75	33.12 ± 2.32	37.96 ± 2.07	34.62 ± 2.02	61.07 ± 5.13	65.25 ± 7.43	3.55 ± 2.05
			RFR	4.53	29.82 ± 1.45	34.51 ± 1.48	30.95 ± 2.11	113.92 ± 3.42	-2.18 ± 3.83	3.79 ± 2.08
			VLL	33.39	34.72 ± 2.29	34.71 ± 2.04	31.92 ± 2.00	90.61 ± 9.71	86.48 ± 10.29	3.56 ± 2.34
			VLR	36.08	30.94 ± 1.58	34.49 ± 2.06	29.80 ± 3.96	111.73 ± 10.6	-12.25 ± 12.04	3.75 ± 2.68
		scan 2								
			RFL	7.46	30.50 ± 2.58	38.27 ± 3.68	36.09 ± 5.96	64.18 ± 7.15	18.92 ± 8.01	3.24 ± 2.66
			RFR	7.75	31.42 ± 10.98	35.27 ± 3.56	31.42 ± 4.37	118.77 ± 3.17	6,20 ± 5.64	3.89 ± 3.44
			VLL	34.22	34.22 ± 2,22	34.33 ± 2.28	31.53 ± 2.23	87.65 ± 7.51	31.48 ± 8.05	2.99 ± 2.70
			VLR	27.38	29.77 ± 4.74	34.59 ± 3.01	28.38 ± 5.12	97.16 ± 14.87	5.05 ± 4.99	3.89 ± 2.77
		scan 3								
			RFL	8.33	31.86 ± 4.52	37.89 ± 3.61	35.72 ± 5.66	67.00 ± 7.19	7.12 ± 6.79	4.18 ± 4.31
			RFR	6.25	30.24 ± 5.19	34.37 ± 2.52	31.12 ± 4.37	119.61 ± 4.16	15.75 ± 7.35	3.39 ± 3.82
			VLL	34.73	31.19 ± 4.58	34.24 ± 2.23	33.10 ± 6.27	92.83 ± 8.52	19.53 ± 9.52	3.48 ± 3.68
			VLR	30.13	29.33 ± 5.93	34.35 ± 2.83	28.98 ± 5.51	98.13 ± 21.09	21.86 ± 7.11	5.28 ± 10.82
**2**	26	scan 1								
			RFL	5.59	32.37 ± 2.79	38.29 ± 2.66	35.14 ± 2.50	66.38 ± 5.70	19.75 ± 6.18	3.36 ± 2.67
			RFR	5.41	31.16 ± 2.07	35.05 ± 3.23	31.15 ± 2.63	115.89 ± 3.77	12.70 ± 4.47	3.39 ± 2.74
			VLL	29.19	33.01 ± 2.62	36.41 ± 3.20	33.19 ± 2.56	88.43 ± 5.52	9.09 ± 7.48	2.72 ± 2.18
			VLR	26.38	32.53 ± 1.73	35.71 ± 2.52	31.14 ± 3.82	105.81 ± 7.28	17.58 ± 3.95	3.43 ± 2.45
		scan 2								
			RFL	6.01	30.81 ± 1.57	37.54 ± 2.72	34.30 ± 2.73	63.71 ± 6.59	10.99 ± 3.80	3.03 ± 2.58
			RFR	5.41	28.80 ± 1.21	34.43 ± 2.99	30.28 ± 2.67	112.02 ± 4.53	9.56 ± 3.15	3.95 ± 2.30
			VLL	29.18	31.47 ± 1.56	35.75 ± 2.92	32.33 ± 2.35	90.81 ± 6.00	5.15 ± 5.02	2.93 ± 2.20
			VLR	27.68	31.17 ± 1.61	35.31 ± 2,72	30.80 ± 3.92	102.38 ± 10.34	10.66 ± 2.80	3.64 ± 2.55
		scan 3								
			RFL	5.13	31.67 ± 2.86	36.38 ± 2.25	33.32 ± 2.13	66.93 ± 6.47	19.87 ± 5.14	4.20 ± 2.60
			RFR	4.93	27.81 ± 2.12	33.66 ± 2.17	29.90 ± 2.70	110.53 ± 5.50	28.08 ± 4.51	3.34 ± 2.78
			VLL	29.28	32.15 ± 2.86	35.10 ± 2.97	32.22 ± 2.71	91.03 ± 9.28	14.19 ± 4.96	2.12 ± 2.17
			VLR	25.89	31.63 ± 2.16	34.74 ± 2.27	30.73 ± 3.59	103.04 ± 7.10	13.20 ± 6.78	3.09 ± 2.54

**3**	24	scan 1								
			RFL	8.27	29.95 ± 1.36	36.04 ± 1.52	32.77 ± 1.50	67.58 ± 5.28	-8.72 ± 3.19	2.55 ± 2.41
			RFR	7.33	29.29 ± 1.19	33.62 ± 1.10	30.09 ± 0.96	115.62 ± 2.76	7.31 ± 5.06	2.69 ± 2.31
			VLL	29.89	31.76 ± 1.52	34.27 ± 1.89	31.64 ± 2.00	89.02 ± 6.24	-11.89 ± 3.03	2.63 ± 2.71
			VLR	28.64	30.56 ± 0.96	33.68 ± 0.86	30.24 ± 2.38	103.43 ± 6.90	11.24 ± 3.21	3.00 ± 2.41
		scan 2								
			RFL	8.47	29.78 ± 1.53	35.63 ± 1.46	32.60 ± 2.03	67.45 ± 5.73	-11.91 ± 2.43	2.39 ± 2.55
			RFR	7.35	28.99 ± 1.16	33.43 ± 1.28	30.45 ± 1.62	114.94 ± 3.46	1.30 ± 4.03	3.01 ± 2.33
			VLL	31.53	30.88 ± 1.66	33.95 ± 2.17	31.11 ± 2.00	90.61 ± 6.34	-10.53 ± 3.24	2.92 ± 2.79
			VLR	29.01	30.19 ± 1.06	33.48 ± 1.02	30.06 ± 2.70	97.95 ± 9.51	9.31 ± 2.15	3.61 ± 2.47
		scan 3								
			RFL	8.39	30.71 ± 1.76	35.51 ± 1.50	32.48 ± 2.00	68.18 ± 4.74	-10.42 ± 2.48	2.58 ± 2.46
			RFR	8.03	29.08 ± 1.41	33.45 ± 1.41	29.91 ± 1.65	117.82 ± 2.95	2.33 ± 4.06	3.01 ± 2.40
			VLL	31.46	30.82 ± 1.92	33.56 ± 2.21	30.70 ± 2.09	92.22 ± 5.84	-9.14 ± 3.55	2.75 ± 2.84
			VLR	27.70	30.17 ± 1.03	33.27 ± 1.07	29.92 ± 2.35	101.31 ± 7.35	9.73 ± 2.12	3.36 ± 2.53
**4**	28	scan 1								
			RFL	7.53	30.11 ± 1.53	32.04 ± 1.81	28.95 ± 1.38	86.91 ± 8.93	19.67 ± 4.83	5.23 ± 2.78
			RFR	7.75	27.32 ± 1.69	32.63 ± 1.87	28.76 ± 2.09	114.28 ± 5.78	16.33 ± 5.38	4.79 ± 2.72
			VLL	41.28	29.25 ± 1.31	33.20 ± 1.54	29.73 ± 1.41	108.76 ± 7.20	17.65 ± 15.04	4.10 ± 2.54
			VLR	42.99	29.56 ± 1.42	32.65 ± 1.75	29.00 ± 2.64	97.69 ± 10.85	3.27 ± 14.31	4.01 ± 2.28
		scan 2								
			RFL	8.56	29.56 ± 2.55	32.41 ± 2.79	29.18 ± 2.22	92.91 ± 8.07	23.29 ± 1.47	4.90 ± 2.81
			RFR	9.50	28.55 ± 1.83	33.36 ± 3.59	29.23 ± 2.71	114.03 ± 5.49	5.86 ± 5.39	5.17 ± 2.97
			VLL	46.11	29.38 ± 1.59	33.02 ± 1.98	29.72 ± 1.62	103.56 ± 13.38	27.80 ± 12.88	3.26 ± 2.24
			VLR	47.78	29.72 ± 1.64	32.82 ± 2.12	29.07 ± 3.12	95.33 ± 9.93	-1.17 ± 13.62	4.41 ± 2.65
		scan 3								
			RFL	6.62	29.76 ± 1.65	31.26 ± 1.40	28.78 ± 2.00	91.14 ± 5.78	29.83 ± 14.69	5.64 ± 4.38
			RFR	7.06	28.80 ± 1.33	31.59 ± 1.34	28.38 ± 1.77	108.87 ± 3.91	8.43 ± 12.02	5.94 ± 2.96
			VLL	41.81	28.68 ± 1.17	32.72 ± 1.15	29.68 ± 1.48	107.34 ± 8.94	28.41 ± 25.51	3.90 ± 3.33
			VLR	43.00	29.43 ± 1.07	32.28 ± 1.54	28.56 ± 2.92	98.50 ± 6.52	3.28 ± 10.41	2.08 ± 3.22
**5**	25	scan 1								
			RFL	6.70	31.55 ± 2.25	35.71 ± 4.01	32.42 ± 2.70	71.15 ± 6.71	-11.21 ± 3.30	4.30 ± 2.28

			RFR	8.88	30.45 ± 3.35	34.91 ± 3.67	30.81 ± 2.65	113.67 ± 5.30	2.11 ± 6.13	4.68 ± 2.68
			VLL	56.89	31.94 ± 2.46	35.04 ± 4.44	31.76 ± 2.81	95.70 ± 9.14	-13.68 ± 10.20	3.64 ± 3.18
			VLR	62.95	32.32 ± 4.08	35.12 ± 3.91	31.23 ± 2.90	93.22 ± 11.36	-5.20 ± 15.31	3.31 ± 2.80
		scan 2								
			RFL	6.85	33.12 ± 3.78	35.77 ± 3.69	32.79 ± 2.79	71.73 ± 6.47	-9.94 ± 5.66	4.60 ± 2.47
			RFR	7.98	32.01 ± 3.58	35.04 ± 3.71	31.67 ± 3.66	117.8 ± 4.61	0.12 ± 3.64	4.75 ± 3.10
			VLL	54.75	32.74 ± 3.45	34.86 ± 3.97	31.95 ± 3.00	99.36 ± 9.98	-13.71 ± 11.77	4.12 ± 3.02
			VLR	59.14	32.67 ± 3.69	34.94 ± 3.83	31.42 ± 3.09	100.32 ± 16.95	-5.83 ± 10.35	3.83 ± 2.82
		scan 3								
			RFL	6.29	33.23 ± 3.04	35.60 ± 3.42	33.33 ± 3.03	70.76 ± 7.80	10.84 ± 10.26	5.50 ± 2.86
			RFR	8.61	32.21 ± 4.27	35.42 ± 3.96	32.13 ± 2.80	108.57 ± 7.21	-13.56 ± 15.27	5.42 ± 2.88
			VLL	53.77	31.81 ± 3.12	34.57 ± 3.88	31.46 ± 3.23	98.61 ± 9.50	6.10 ± 19.81	4.30 ± 3.10
			VLR	60.61	32.14 ± 3.73	34.73 ± 3.74	31.45 ± 3.26	97.74 ± 17.02	-15.25 ± 13.10	3.81 ± 2.86
**6**	29	scan 1								
			RFL	7.47	29.30 ± 1.44	35.46 ± 2.26	32.09 ± 1.59	63.86 ± 6.09	3.89 ± 6.47	3.68 ± 2.54
			RFR	7.88	27.28 ± 1.46	32.87 ± 2.47	29.12 ± 1.87	113.05 ± 3.79	5.69 ± 7.81	4.24 ± 2.29
			VLL	46.13	29.68 ± 1.12	33.29 ± 1.98	30.28 ± 1.89	92.31 ± 7.68	2.18 ± 9.68	3.17 ± 2.52
			VLR	46.92	29.06 ± 1.03	33.29 ± 1.93	29.56 ± 2.23	97.03 ± 10.04	4.47 ± 11.76	3.58 ± 2.68
		scan 2								
			RFL	7.25	30.16 ± 1.56	35.64 ± 1.91	32.71 ± 1.76	61.63 ± 6.98	1.91 ± 9.46	4.81 ± 2.61
			RFR	7.03	29.30 ± 1.50	33.17 ± 1.71	29.69 ± 1.62	112.84 ± 3.35	0.12 ± 5.87	4.11 ± 2.31
			VLL	45.71	30.71 ± 1.26	33.89 ± 1.55	31.05 ± 1.88	91.57 ± 9.24	4.90 ± 7.47	3.02 ± 2.45
			VLR	43.14	30.58 ± 1.15	33.52 ± 1.49	29.99 ± 2.47	101.95 ± 9.03	3.84 ± 6.99	3.86 ± 2.59
		scan 3								
			RFL	5.51	30.46 ± 1.32	34.88 ± 1.54	31.64 ± 1.25	69.07 ± 6.78	6.42 ± 8.89	4.60 ± 2.22
			RFR	6.45	28.88 ± 1.16	32.97 ± 1.51	29.30 ± 1.49	114.62 ± 3.48	-1.21 ± 4.44	3.95 ± 2.42
			VLL	40.80	30.35 ± 1.22	33.29 ± 1.27	30.37 ± 1.74	93.33 ± 8.23	4.66 ± 9.14	2.75 ± 2.33
			VLR	43.91	29.74 ± 1.03	33.32 ± 1.58	29.38 ± 2.69	101.35 ± 11.86	2.79 ± 6.54	3.63 ± 2.51
**7**	22	scan 1								
			RFL	10.33	30.81 ± 2.97	35.36 ± 2.82	32.21 ± 2.29	69.74 ± 11.54	9.73 ± 11.47	4.18 ± 2.24
			RFR	11.88	29.36 ± 1.48	33.26 ± 1.37	29.93 ± 1.36	115.75 ± 2.93	0.36 ± 3.93	3.26 ± 2.52
			VLL	34.02	31.30 ± 2.49	33.49 ± 2.28	30.97 ± 2.51	94.83 ± 4.74	24.35 ± 9.43	3.58 ± 3.39

			VLR	32.70	31.05 ± 3.73	33.42 ± 2.83	29.66 ± 3.52	98.24 ± 5.83	10.91 ± 8.34	4.48 ± 3.25
		scan 2								
			RFL	11.58	30.89 ± 2.67	35.28 ± 3.64	32.08 ± 2.63	73.22 ± 11.12	20.99 ± 7.22	3.77 ± 2.10
			RFR	13.75	28.68 ± 1.83	33.55 ± 2.84	29.78 ± 1.70	115.69 ± 4.37	2.12 ± 4.23	3.67 ± 2.44
			VLL	34.97	31.21 ± 2.99	34.81 ± 4.61	31.21 ± 3.27	91.52 ± 6.23	30.52 ± 7.51	3.12 ± 3.46
			VLR	34.70	31.01 ± 2.71	34.65 ± 5.05	30.53 ± 4.49	95.84 ± 5.86	7.11 ± 11.1	4.63 ± 3.31
		scan 3								
			RFL	10.95	30.92 ± 2.39	35.66 ± 3.28	32.50 ± 2.76	67.77 ± 9.80	29.93 ± 14.86	4.10 ± 2.27
			RFR	12.91	27.99 ± 1.93	32.96 ± 1.74	29.45 ± 1.81	115.29 ± 3.97	-2.30 ± 7.24	4.08 ± 2.62
			VLL	33.88	31.87 ± 2.88	33.33 ± 2.58	30.47 ± 2.38	92.29 ± 4.63	36.83 ± 10.08	3.96 ± 3.63
			VLR	35.10	31.42 ± 3.01	33.11 ± 3.00	29.14 ± 3.98	97.61 ± 10.32	2.86 ± 10.47	3.80 ± 3.24
**8**	27	scan 1								
			RFL	7.84	31.36 ± 1.76	35.40 ± 2.11	32.63 ± 1.75	66.99 ± 7.86	14.44 ± 5.53	3.41 ± 2.46
			RFR	9.66	29.71 ± 1.45	34.20 ± 1.97	30.47 ± 1.67	115.55 ± 5.52	9.70 ± 5.17	3.20 ± 3.09
			VLL	31.59	31.67 ± 1.71	34.57 ± 2.44	31.80 ± 2.07	99.61 ± 10.99	16.59 ± 7.68	4.57 ± 2.75
			VLR	29.35	31.57 ± 1.75	34.31 ± 2.17	30.73 ± 2.89	92.52 ± 21.29	1.14 ± 10.53	5.23 ± 2.81
		scan 2								
			RFL	8.79	29.13 ± 3.12	33.70 ± 1.71	30.48 ± 1.43	72.54 ± 8.32	-10.06 ± 1.95	3.27 ± 2.39
			RFR	10.33	27.14 ± 2.58	33.46 ± 1.81	29.09 ± 1.38	118.06 ± 5.38	-0.39 ± 3.22	3.26 ± 2.73
			VLL	35.72	28.81 ± 3.07	33.48 ± 2.53	30.41 ± 2.12	94.82 ± 16.76	-10.46 ± 4.05	4.73 ± 4.13
			VLR	31.10	28.55 ± 1.73	33.42 ± 1.71	29.18 ± 3.42	89.72 ± 13.56	-3.03 ± 6.90	4.66 ± 2.86
		scan 3								
			RFL	8.22	32.29 ± 3.51	34.15 ± 2.02	30.51 ± 1.59	67.73 ± 9.01	-4.64 ± 4.64	2.64 ± 2.5
			RFR	9.97	27.10 ± 2.90	33.52 ± 2.00	28.75 ± 1.71	116.55 ± 7.32	-6.97 ± 5.63	2.76 ± 2.92
			VLL	28.81	30.44 ± 3.47	33.48 ± 1.77	29.98 ± 2.01	93.22 ± 12.33	-2.35 ± 6.62	4.67 ± 2.65
			VLR	29.25	31.50 ± 3.88	33.77 ± 1.92	29.37 ± 2.97	109.40 ± 19.41	-2.60 ± 6.96	4.83 ± 3.52
**9**	21	scan 1								
			RFL	12.89	32.02 ± 2.01	35.80 ± 3.58	32.67 ± 3.14	69.84 ± 9.29	20.53 ± 3.26	3.70 ± 3.27
			RFR	11.74	28.40 ± 1.45	34.19 ± 2.62	30.00 ± 2.98	120.70 ± 5.07	-10.76 ± 4.76	3.71 ± 3.38
			VLL	42.93	32.20 ± 1.58	35.72 ± 3.11	32.87 ± 2.54	97.36 ± 9.15	12.88 ± 4.29	5.70 ± 3.05
			VLR	45.16	33.23 ± 2.06	35.57 ± 2.54	31.89 ± 2.86	95.76 ± 12.38	-39.83 ± 12.34	5.51 ± 3.06
		scan 2								

			RFL	13.86	32.29 ± 3.35	36.71 ± 4.42	33.28 ± 4.33	69.90 ± 12.05	29.52 ± 2.41	3.18 ± 3.50
			RFR	12.78	28.01 ± 2.58	34.47 ± 2.95	30.03 ± 3.21	116.79 ± 8.04	-11.57 ± 5.57	3.12 ± 3.45
			VLL	47.22	31.89 ± 1.62	35.80 ± 3.20	32.25 ± 2.42	98.53 ± 8.49	28.33 ± 4.24	4.73 ± 3.37
			VLR	50.37	33.20 ± 2.27	35.97 ± 3.13	31.86 ± 3.56	93.84 ± 14.10	-38.96 ± 10.01	5.24 ± 3.48
		scan 3								
			RFL	16.02	32.37 ± 3.80	36.53 ± 4.23	33.39 ± 4.25	69.16 ± 9.77	17.94 ± 4.05	3.77 ± 3.72
			RFR	13.34	28.30 ± 1.95	34.24 ± 2.83	29.96 ± 3.04	114.91 ± 7.63	-9.10 ± 5.61	3.32 ± 3.53
			VLL	51.59	32.27 ± 2.26	35.66 ± 3.37	32.56 ± 2.84	95.63 ± 10.06	15.62 ± 4.84	5.24 ± 3.91
			VLR	47.19	32.95 ± 2.18	35.39 ± 2.91	31.56 ± 2.99	85.38 ± 19.20	-36.06 ± 11.09	5.53 ± 3.47
**10**	22	scan 1								
			RFL	6.37	32.11 ± 4.15	42.52 ± 7.56	38.77 ± 6.66	58.02 ± 7.08	-18.36 ± 2.47	12.79 ± 15.81
			RFR	8.65	27.19 ± 1.30	33.66 ± 1.79	29.89 ± 2.15	114.67 ± 5.52	18.99 ± 2.55	3.04 ± 2.93
			VLL	26.29	31.24 ± 1.21	33.74 ± 1.54	30.51 ± 1.96	94.75 ± 8.14	-29.69 ± 12.35	4.67 ± 3.07
			VLR	28.97	30.41 ± 1.88	33.70 ± 2.48	29.48 ± 3.62	99.75 ± 16.90	16.93 ± 9.00	24.82 ± 3.98
		scan 2								
			RFL	2.81	37.18 ± 6.49	46.92 ± 8.13	42.44 ± 7.11	57.16 ± 7.33	-18.69 ± 3.81	15.19 ± 15.13
			RFR	6.69	27.17 ± 1.30	33.95 ± 1.66	29.91 ± 2.19	114.08 ± 3.90	20.43 ± 3.99	2.67 ± 2.91
			VLL	24.01	32.26 ± 1.70	34.04 ± 2.15	30.75 ± 2.09	97.71 ± 7.62	-34.67 ± 12.92	4.68 ± 3.05
			VLR	23.18	30.47 ± 2.01	33.89 ± 2.33	29.32 ± 3.91	107.25 ± 19.41	29.19 ± 6.31	5.25 ± 3.48
		scan 3								
			RFL	4.80	33.62 ± 3.86	41.99 ± 6.12	38.41 ± 6.36	54.18 ± 7.53	-19.42 ± 2.29	8.80 ± 12.23
			RFR	8.25	28.20 ± 1.77	34.28 ± 2.25	33.12 ± 10.22	113.39 ± 4.13	11.58 ± 3.41	2.23 ± 2.85
			VLL	29.75	31.92 ± 1.59	34.54 ± 2.39	31.06 ± 1.77	90.90 ± 7.51	-29.79 ± 9.85	4.54 ± 3.22
			VLR	27.67	30.39 ± 2.49	34.19 ± 2.75	29.54 ± 4.06	98.93 ± 13.74	9.24 ± 10.61	4.49 ± 3.45
